# Discourse intervention strategies in Alzheimer's disease: Eye-tracking and the effect of visual cues in conversation

**DOI:** 10.1590/S1980-57642014DN83000012

**Published:** 2014

**Authors:** Lenisa Brandão, Ana Maria Monção, Richard Andersson, Kenneth Holmqvist

**Affiliations:** 1 Professor in Speech-Language Pathology, Departamento de Saúde e Comunicação Humana, Instituto de Psicologia, Universidade Federal do Rio Grande do Sul, RS, Brazil.; 2 Professor in Linguistics, Centro de Linguística, Universidade Nova de Lisboa, Lisbon, Portugal.; 3 Doctor in Cognitive Science, Humanities Lab, Lund University, Lund, Sweden.; 4 Professor in Psychology, Humanities Lab, Lund University, Lund, Sweden.

**Keywords:** Alzheimer's disease, discourse, attention, visual cues

## Abstract

**Objective:**

The goal of this study was to investigate whether on-topic visual cues can serve as aids for the maintenance of discourse coherence and informativeness in autobiographical narratives of persons with Alzheimer's disease (AD).

**Methods:**

The experiment consisted of three randomized conversation conditions: one without prompts, showing a blank computer screen; an on-topic condition, showing a picture and a sentence about the conversation; and an off-topic condition, showing a picture and a sentence which were unrelated to the conversation. Speech was recorded while visual attention was examined using eye tracking to measure how long participants looked at cues and the face of the listener.

**Results:**

Results suggest that interventions using visual cues in the form of images and written information are useful to improve discourse informativeness in AD.

**Conclusion:**

This study demonstrated the potential of using images and short written messages as means of compensating for the cognitive deficits which underlie uninformative discourse in AD. Future studies should further investigate the efficacy of language interventions based in the use of these compensation strategies for AD patients and their family members and friends.

## Abstract (pt)

**Objetivo:**

O objetivo deste estudo foi o de investigar se pistas visuais sobre o tópico discursivo podem servir como auxiliares para a manutenção da coerência e da informatividade em narrativas autobiográficas de pessoas com doença de Alzheimer (DA).

**Métodos:**

O experimento apresentou três condições: uma conversa tendo à vista uma tela de computador em branco; uma condição de conversação com pista dentro do tópico, em que a tela do computador mostrava uma foto e uma frase sobre o evento tema; e uma condição fora de tópico, em que a tela mostrava uma foto e uma frase que não apresentavam relação com o tema da conversa. O discurso foi gravado enquanto a atenção visual foi examinada através de rastreamento ocular para medir a atenção dos participantes para as pistas apresentadas na tela e para o rosto do ouvinte.

**Resultados:**

Os resultados sugerem que as intervenções por meio de pistas visuais na forma de imagens e informações escritas são úteis para melhorar a informatividade discursiva na AD.

**Conclusão:**

Este estudo demonstrou o potencial do uso de imagens e mensagens escritas curtas como meios de compensação para aumentar a informatividade do discurso em pacientes com DA. Estudos futuros devem investigar a eficácia de intervenções de linguagem baseadas no uso dessas estratégias de compensação para a comunicação de pacientes com DA com seus familiares e amigos.

## INTRODUCTION

The use of external aids to improve the conversational skills of patients with Alzheimer's disease (AD) were first reported in the 1990s.^2-3^ Studies on memory wallets seem to have been discontinued, but research on the use of recipe books with images and short sentences has suggested that visual information facilitates access to memory.^4^ These authors emphasized the need for future studies to investigate the effect of relevant visual stimuli in the discourse production of AD patients.

Eye tracking is widely used to investigate visual perception and attention during different cognitive and linguistic tasks.^5^ However, eye-tracking studies with AD participants have not focused specifically on the communication skills of this population. Instead, there has been an emphasis on demonstrating impairment in a range of cognitive abilities related to visual attention.^6^ However, there is evidence that persons with AD are able to successfully focus their attention on targets flanked by distractors on a screen.^7^ Visual orientation and sustained attention to emotionally-arousing scenes are also preserved in AD.^8^

Nevertheless, there is evidence that, as AD progresses, a loss of interest in the listener can be observed.^9^ Monitoring gaze direction provides information about the listener's engagement in or disengagement from tasks that require shared attention.^10^ Attention to faces seems to be reduced in this population. A study about visual attention to faces demonstrated that AD patients dwelled on faces in pictures for shorter periods of time than the control group.^11^ Patients in the severe stage of AD may not lose their ability to care about the nonverbal reaction of their communication partners,^12^ but whether attention to faces is well preserved in communication contexts has yet to be investigated.

The goal of the present study was to investigate the discourse and visual attention of AD participants and healthy older adults to on- and off-topic cues. The hypothesis was that participants with AD produce a more coherent and informative discourse while fixating on-topic visual cues.

## METHOD

**Participants.** Participants comprised:

(a) a control group of 10 older adults without dementia, who attended the Universidade Senior de Oeiras; and
(b) five older adults with moderate-stage AD, who were outpatients of the Clínica São José located in Lisbon. The selection of participants with AD was performed by two neurologists of the Clínica São José (Lisbon, Portugal), who established the diagnosis of probable Alzheimer's dementia.^13^

The control group comprised predominantly women (70%); and, in the AD group, three out of five participants were women. The mean age of the control group was 78.31 years (6.65). In the AD group, the mean age was 80.92 (5.51) (U=89.00; p=0.35). In terms of educational level, there were no significant differences between groups (control: 6.12 (1.58); AD: 6.00 (1.66); U=108.00; p=0.88). The exclusion criteria of dementia for controls were: score ranging from 27 to 30 on the Mini-Mental State Examination (MMSE) (14; 15); autonomy in daily life; and absence of cognitive complaints, neurological, and psychiatric disorders based on data provided in an interview about health-related and sociodemographic data. The mean score of controls on the MMSE was 28.37 (1.02). The mean score of participants with AD on the MMSE differed significantly from the control sample (M=20.91; SD=4.25; p<0.05). Most of the participants with AD had moderate decline (MMSE=16). The study was approved by the Research Ethics Committee of Clínica São José in Portugal, where data collection took place. Participants included in the AD group and their relatives, as well as the control group, all signed a written consent form explaining the tasks planned for the study.

**Study design.** This was a cross-sectional, quasi-experimental study, based on mixed analysis using comparison between groups, cases and controls, as well as comparison between conditions. Three different conditions were used:

[A] conversations during which on-topic visual cues were displayed on a screen,
[B] conversations during which off-topic visual cues were displayed on a screen, and
[C] conversations during which a blank screen was displayed. All conditions were focused on the participants' personal reports about their youth years (between 20 and 30 years old). A family member of each participant previously selected two important events in the participant's life (one to be used in conditions A and B and the other for condition C). Family members were sent a confidential letter containing instructions on how to select pictures and create sentences to be used in the study.

Youth years were established based on studies demonstrating special focus on autobiographical memory during this period, which is known as the memory bump for older adults.^17^ The pictures and sentences provided by family members were reviewed and selected to decide which event would be told during the experiment. The same event was used in conditions A and B to keep memory differences from interfering with the results of visual attention and discourse. Because of this, the events were used in a randomized order to prevent effects of practice from interfering with the results. Two researchers participated in the conversation experiment to ensure that the participants did not report the event twice to the same listener, which would increase context artificiality. A different important life event was used in condition C to avoid excessive effects of practice. With this in mind, each examiner's participation was balanced to avoid greater participation by any one of the examiners in a given condition, which could also interfere with the differences between the conditions. Discourse data of condition C were used to provide a means of comparison between the samples in a condition without visual cues.

Pictures were chosen so that the image cues of conditions A and B were not significantly different in terms of those visual characteristics that tend to have an influence on the fixation time of pictures, such as number of people in the picture, size of the faces, presence of children and animals, and emotional arousal (pleasantness and facial expression arousal). Characteristics such as color, brightness, and size of the pictures were controlled using the computer program *Photoshop* by Microsoft, so that all characteristics were similar in conditions A and B. The sentences provided by family members were revised to be simple sentences containing approximately the same number of words and relevant information in order to be similar to the title of the event. The schedule of the experiment was set using the computer program* E-prime.*^18^

**Procedures.** A mobile, head-mounted eye tracker (SMI HED 50Hz incl. Polhemus head tracking) was used for data collection. Adjustments were made aimed at placing the camera and the lens in a position to capture appropriate images of the eye, including the analysis of corneal and pupillary reflexes on a computer used to record eye-tracking data. Describing the procedures prevented the AD participants from being afraid of wearing the reflective lens, which was positioned at a safe distance from the eye. Once an appropriate image was achieved, calibration was done.

After calibration, the researcher pressed the key to start the presentation of the instructions on the screen, while the first examiner entered the room. The examiner sat facing the participant and explained the instructions that were being displayed on the screen:

Next, you'll be asked to tell me about certain life events. Please tell the communicative partner sitting in front of you about the life event suggested. A picture and a sentence about the event might be displayed on the screen, or a picture and a sentence about a different event might be displayed, or the screen might be blank. Feel free to look at the screen for as long as you want while you are talking about the event. The next interviewer will not listen to your report. The second interviewer will substitute the first one and you will be asked to tell the same story again.

Each participant was randomly exposed to the three conditions. In all the conditions, the picture and the sentence were displayed on the screen during the period of time each participant took to complete the story. Examiners were graduate students trained to limit their participation to certain speech acts during the conversation:

(a) offering signs of attention, interest, and emotional reactions;
(b) when participants interrupted their discourse, examiners provided verbal clues such as "What else"; and
(c) when participants switched topic, the examiner provided a verbal clue to lead back to the topic.

**Data analysis.** Total fixation time, known as ‘dwell time'^19^ on regions of interest (picture, sentence, and interlocutor's face) was analyzed by checking possible differences between samples and between conditions.

All discourse samples were transcribed verbatim and segmented into propositions. The coded variables were analyzed using the computer program *CHAT* of the *CHILDS* project.^20^ Discourse was divided into propositions and classified in terms of global coherence and informativeness according to a method adapted from Laine, Laakso, Vuorinen and Rinne.^21^ Twenty percent of the corpus was randomly selected and coded by two examiners, a speech therapist and a linguist blind to the conditions and to the group of participants. The Kappa test was used to evaluate the reliability of the discursive analysis. Global coherence showed 77% agreement; whereas informativeness had 81% agreement. According to Linell, Gustavsson and Juvonen,^22^ a 75% agreement is realistic for a conversation analysis, considering that interaction is often obscured by ambiguity. Carletta^23^ recommends that Kappa agreement should not be < 0.67 in order to allow reliable conclusions.

**Statistical analysis.** Because of the objective characteristic of the eye-tracking data, small groups were compared using non parametric tests with the statistical package SPSS.^24^ The Mann-Whitney test was used to compare the groups, whereas the Wilcoxon test was applied to compare the visual tracking data between each of the conditions (within each sample). Regarding discursive data, each of the AD cases was compared with the control group to ensure a more accurate analysis. For this purpose, a special statistical program (SINGLIMS.EXE) was used for the comparison of cases with a small control group. According to this modified t-test,^25^ t values suggested significant differences between each AD case and the control group. The closer to zero the t values, the greater the likelihood of proving the null hypothesis that the case is part of the control group.

## RESULTS

AD patients looked longer (ms) at the sentence on the screen than controls, both in the condition displaying on-topic visual cues (U=54.50; p<0.01) and in the condition showing off-topic visual cues (U=65.00; p<0.05). With regard to the total fixation time on the interlocutor's face, we found a statistically significant difference between the groups in the off-topic condition: the control group looked longer at the interlocutor's face than AD participants (U=62.50; p<0.05). This was not true in the on-topic condition (U=71.00; p=0.09). As regards the total fixation time on the picture, there were no significant differences between the groups both in the on-topic condition (U=91.00; p=0.4) and in the off-topic condition (U=109.0; p=0.91). In addition to fixating the listener's face for a longer period of time in the off-topic condition, the control group also looked longer at the screen than AD participants in the blank screen condition (U=64.50; p<0.05).

The analysis of the differences between fixation time on the screen and fixation time on the face revealed that the control group demonstrated fixation time on the face that was not statistically different from fixation time on the screen (Z= -1.86; p=0.06), although it was longer. Conversely, in the AD group, this difference was significant because AD participants looked longer at the listener's face than at the screen (Z= -2.35; p<0.01). Descriptive data are shown in Table 1.

**Table 1:** Total fixation time on visual input in each condition.

Visual input	Condition	Control group Mean (SD)	AD group Mean (SD)
Picture	On-topic	51955.70 (46684.06)	28525.63 (18830.30)
	Off-topic	25145.24 (37429.97)	18020.18 (17869.98)
Sentence	On-topic	929.50 (1628.17)	4211.30 (5987.18)
	Off-topic	931.99 (1675.70)*	4108.49 (5718.17)*
Interlocutor’s face	On-topic	34476.99 (37341.64)	29880.66 (63644.09)
	Off-topic	49384.57 (45755.71)*	21510.70 (32514.94)*
	Blank screen	60248.32 (74996.44)	25245.84 (24115.81)
Screen	On-topic	52885.20 (47352.45)	32736.93 (21938.80)
	Off-topic	26069.16 (38781.59)	21485.82 (23065.10)
	Blank screen	22566.28 (37513.37)*	2925.24 (3587.05)*

* Represents significant difference between the groups, p<0.05.

The control group looked for a significantly longer time at the screen in the on-topic condition than in the off-topic condition (Z= -2.53; p<0.01). Although the AD group showed a tendency to look longer at the screen in the on-topic condition than in the off-topic condition, the difference between the conditions was not significant (Z= -1.85; p=0.06). When comparing the on-topic and off-topic conditions with the blank screen condition, the control group was found to have looked longer at the screen in the on-topic condition than in the blank screen condition (Z= -2.66; p<0.01). This was not true for the control group when the blank screen condition was compared with the off-topic condition (Z= -0.90; p=0.36). Conversely, the AD group looked longer at the screen in both conditions including cues, with significant differences when comparing the blank screen condition both with the on-topic condition (Z= -3.29; p<0.01) and with the off-topic condition (Z= -2.66; p<0.01). Therefore, the AD group paid much more attention to the screen in the conditions displaying visual cues than in the blank screen condition, showing particular interest in the cues on the screen, regardless of their relevance to the context of the discourse.

When comparing the total fixation time on the interlocutor's face, the control group showed a tendency to look longer at the interlocutor's face in the blank screen condition than in the conditions displaying on-topic and off-topic cues. However, this difference was not significant (χ^2^=5.2; p=0.07). AD participants did not show this tendency, having similar fixation times on the face under all three conditions (χ^2^=1.28; p=0.52).

In the control group, the picture was the cue at which the participants looked longer, having looked much longer at the on-topic picture than at the off-topic picture (Z= -2.63; p<0.01). The picture was also the cue at which the AD group looked longer; however, in this group, there were no significant differences between the fixation time on the picture for on-topic and off-topic conditions (Z= -1.6; p=0.1). With regard to the total fixation time for the sentence, there were no differences between the conditions both in the control group (Z= -0.03; p=0.97) and in the AD group (-0.59; p=0.55). The fixation time for the listener's face also showed no significant difference between the conditions in the control group (Z= -1.59; p=0.11) and in the AD group (Z= -0.91; p=0.36).

Two AD participants had significantly lower global coherence scores than controls in the on-topic condition. The discourse of the other three AD participants did not differ from that of controls in terms of global coherence under the same condition. Conversely, in the off-topic condition and in the blank screen condition, the discourses of the three AD participants were significantly less coherent than that of controls (Table 2).

**Table 2:** Differences between cases and controls in global coherence scores.

Participants AD cases	On-topic condition Score	Off-topic condition Score	Blank screen condition Score
A	83.45	57.27*	54.10*
B	43.22*	21.87*	32.71*
C	70.20	65.15	71.31
D	87.23	66.66	52.23
E	46.36*	28.57*	34.29*
M (SD) controls	80.55 (15.62)	82.34 (14.85)	80.20 (11.35)

Differences between each AD case and the control group are marked for p<0.05. P values indicate an estimated percentage of the control population that would have lower scores than the cases.

As shown in Table 3, in terms of informativeness, all AD participants had similar scores to those of controls in the on-topic condition. Conversely, in the off-topic condition, the discourses of two AD participants were significantly less informative than those of controls. However, three AD participants had similar scores of informativeness to those of controls in this condition. In the blank screen condition, three AD participants demonstrated significantly less informative discourses than controls.

**Table 3:** Differences between cases and controls in terms of informativeness

Participants AD cases	On-topic condition Score	Off-topic condition Score	Blank screen condition Score
A	84.25	65.24	26.33*
B	67.42	29.71*	35.30*
C	76.32	70.33	73.11
D	82.54	72.12	84.37
E	63.22	39.37*	47.14*
M (SD) controls	86.49 (16.53)	82.91 (11.96)	85.27 (15.10)

Differences between each AD case and the control group are marked for p<0.05. P values indicate an estimated percentage of the control population that would have lower scores than the cases.

## DISCUSSION

In general, the results of this study are in agreement with, and can be understood in the light of, related findings from studies investigating visual attention and discourse abilities of individuals with AD and healthy controls. Concerning visual attention, eye-tracking measures showed that, unlike the control group, AD participants did not differ in the time spent dwelling for on- and off-topic cues. This finding is in agreement with studies showing that the inhibition deficits of patients with AD affect their ability to disengage attention from irrelevant stimuli.^26^ AD participants paid attention to visual cues, but did not have sufficiently preserved attentional skills to independently choose to focus exclusively on relevant visual stimuli. This result demonstrates that individuals with AD may benefit from interventions conducted by therapists and family members trained to select and display only those cues directly relevant to the context of conversations. According to Bourgeois,^3^ discourse interventions designed for AD patients should consider that direct external aids are more effective than self-monitored strategies.

Eye-tracking data demonstrated that when the screen was displaying irrelevant information, controls looked instead at the examiner's face. On the other hand, in the blank screen condition, controls used the gaze averting strategy by looking more frequently to the screen. This did not occur with AD participants, who were much more attracted to the listener's face in the blank screen condition. This condition required participants to recall and recount an event without the display of visual cues that could possibly help them. It can be said that this situation demands greater independence and the ability of the speaker to rely on his or her own resources in order to retrieve information for discourse processing. Since the classic study of Argyle and Cook,^27^ it is known that during complex discourse planning, healthy adults frequently avoid looking at the listener's face in an attempt to focus on information processing. AD participants rarely looked at the screen in this task and were drawn to their listener's face for a much longer period, possibly in an attempt to obtain help and as a strategy for switching turns. This finding reinforces the idea that AD participants rely greatly on their communicative partners. In fact, strategies in which AD speakers attempt to obtain the listener's help and try to switch turns confirm the preservation of certain pragmatic abilities in moderate and moderate-severe stages of AD.^28^ Looking longer at the listener's face also confirms the idea that in moderate stages of AD there is no significant decrease in interest in the interlocutor.^9^ There is evidence that important non-verbal pragmatic abilities necessary for enjoying the context of communication are present even in the latest stages of AD.^12^ Thus, independently of the severity of AD and of the difficulties in communicating verbally, there is undoubtedly interest and need for communication.

Although pictures were the most focused cues, written information was dwelled on much longer by AD participants than by controls. Mahendra and Bayles^29^ demonstrated that written visual information is more efficient as a mnemonic cue for AD patients than auditory information. The authors argued that, whereas sentences produced by an interlocutor disappear from an AD person's working memory, written sentences can be read again many times as a mnemonic aid while performing a task. Therefore, it is possible that the AD group in the present study looked longer at the written information in an attempt to use it as a retrieval aid and a cue to keep up with the conversation.

We found no significant differences between the discourse of the participants with AD and controls in terms of informativeness in the on-topic condition. Conversely, in the condition including off-topic cues and in the blank screen condition, the discourse of two and three cases, respectively, was significantly less informative and less coherent. These findings should be regarded with caution given the heterogeneity of the patterns demonstrated by the cases in each condition. Due to their very nature, this kind of data tend to show more individual differences, but the heterogeneity of discourse results was probably greater because of the small sample size, which represents a limitation of the present study. Nevertheless, most of the results are in agreement with previous findings^3^ demonstrating that individuals with AD are able to take advantage of visual cues to express more factual information and fewer ambiguous and repetitive sentences. The advantage of using autobiographical visual cues may be that they seem to work as sensory aids that do not require cognitive effort. Dijkstra et al.^4^ suggested that the visual information provided by recipe books facilitated access to semantic memory. With regard to the discursive task proposed in the present study, visual cues seemed to help AD participants to access information from autobiographical memory and to produce new propositions. It is possible that the impaired executive system responsible for retrieving relevant autobiographical information is benefited by the use of a direct channel created by visual input based on implicit activation mechanisms that do not require great cognitive effort. Taken together, our results suggest that on-topic pictures and short sentences may be helpful in prompting AD participants to activate relevant ideas in conversations. Future studies should investigate the efficacy of such interventions on a larger scale.

**Acknowledgements.** The first received Post-doctorate scholarship awarded by the Portuguese Science and Technology Foundation (*Fundação para a Ciência e Tecnologia - FCT*) for the execution of this study at the Center of Linguistics of Universidade Nova de Lisboa. The authors would like to thank the collaboration of the senior university of Oeiras (*Universidade Senior de Oeiras*) and its students, who enthusiastically volunteered as controls. We also thank the participants with AD and their family members, as well as Dr. Manuel Goncalves Pereira for his kind collaboration in recruiting the patients from the Clinica Sao Jose.
